# Week 48 Resistance Analyses of the Once-Daily, Single-Tablet Regimen Darunavir/Cobicistat/Emtricitabine/Tenofovir Alafenamide (D/C/F/TAF) in Adults Living with HIV-1 from the Phase III Randomized AMBER and EMERALD Trials

**DOI:** 10.1089/aid.2019.0111

**Published:** 2019-12-31

**Authors:** Erkki Lathouwers, Eric Y Wong, Kimberley Brown, Bryan Baugh, Anne Ghys, John Jezorwski, El Ghazi Mohsine, Erika Van Landuyt, Magda Opsomer, Sandra De Meyer

**Affiliations:** ^1^ Janssen Pharmaceutica NV, Beerse, Belgium.; ^2^ Janssen Scientific Affairs, LLC, Titusville, New Jersey.; ^3^ Janssen Research & Development LLC, Raritan, New Jersey.; ^4^ Janssen Research & Development, Pennington, New Jersey.

**Keywords:** darunavir/cobicistat/emtricitabine/TAF, single-tablet regimen, resistance, efficacy, deep sequencing, archived RAMs

## Abstract

Darunavir/cobicistat/emtricitabine/tenofovir alafenamide (D/C/F/TAF) 800/150/200/10 mg is being investigated in two Phase III trials, AMBER (NCT02431247; treatment-naive adults) and EMERALD (NCT02269917; treatment-experienced, virologically suppressed adults). Week 48 AMBER and EMERALD resistance analyses are presented. Postbaseline samples for genotyping/phenotyping were analyzed from protocol-defined virologic failures (PDVFs) with viral load (VL) ≥400 copies/mL at failure/later time points. *Post hoc* analyses were deep sequencing in AMBER, and HIV-1 proviral DNA from baseline samples (VL <50 copies/mL) in EMERALD. Through week 48 across both studies, no darunavir, primary PI, or tenofovir resistance-associated mutations (RAMs) were observed in HIV-1 viruses of 1,125 participants receiving D/C/F/TAF or 629 receiving boosted darunavir plus emtricitabine/tenofovir-disoproxil-fumarate. In AMBER, the nucleos(t)ide analog reverse transcriptase inhibitor (N(t)RTI) RAM M184I/V was identified in HIV-1 of one participant during D/C/F/TAF treatment. M184V was detected pretreatment as a minority variant (9%). In EMERALD, in participants with prior VF and genoarchive data (*N* = 140; 98 D/C/F/TAF and 42 control), 4% had viruses with darunavir RAMs, 38% with emtricitabine RAMs, mainly at position 184 (41% not fully susceptible to emtricitabine), 4% with tenofovir RAMs, and 21% ≥ 3 thymidine analog-associated mutations (24% not fully susceptible to tenofovir) detected at screening. All achieved VL <50 copies/mL at week 48 or prior discontinuation. D/C/F/TAF has a high genetic barrier to resistance; no darunavir, primary PI, or tenofovir RAMs were observed through 48 weeks in AMBER and EMERALD. Only one postbaseline M184I/V RAM was observed in HIV-1 of an AMBER participant. In EMERALD, baseline archived RAMs to darunavir, emtricitabine, and tenofovir in participants with prior VF did not preclude virologic response.

## Introduction

With the increasing choice of combination HIV-1 antiretroviral therapy (ART) regimens with similar high efficacy, selection of an ART regimen is frequently based on safety, tolerability, convenience, and genetic barrier to ART resistance.^1–3^ Understanding the resistance profile of the component antiretrovirals and the potential for emergence of resistance-associated mutations (RAMs) is important, as this is likely to influence the extent of treatment and future treatment options.^1–3^

Since its approval in 2006, a wealth of clinical trial data and clinical experience has been generated for boosted darunavir (DRV), demonstrating its durable virologic response, high genetic barrier to resistance, and long-term safety in a broad range of patients from ART-naive to highly treatment experienced.^4–11^ The once-daily, single-tablet regimen (STR) of darunavir/cobicistat/emtricitabine/tenofovir alafenamide (D/C/F/TAF) 800/150/200/10 mg is approved in the United States, Europe, and Canada^12,13^ and is being investigated in two international, randomized Phase III trials, AMBER in ART-naive adults^10^ and EMERALD in ART-experienced, virologically suppressed adults living with HIV-1.^11^ D/C/F/TAF had noninferior efficacy versus D/C plus emtricitabine/tenofovir disoproxil fumarate (F/TDF) (week 48 virologic response, viral load [VL] <50 copies/mL: 91.4% vs. 88.4%, respectively; FDA-snapshot analysis) in AMBER^10^ and versus boosted protease inhibitor (bPI) + F/TDF for the primary efficacy outcome of protocol-defined virologic rebound (PDVR) cumulative through week 48 in EMERALD (D/C/F/TAF: 2.5% vs. bPI + F/TDF: 2.1%).^11^ Week 48 virologic failure (VF, FDA snapshot; VL ≥50 copies/mL) was 0.8% versus 0.5%, respectively, and virologic response was 94.9% versus 93.7%.^11^ Current treatment guidelines include DRV and cobicistat combined with two nucleoside or nucleotide analog reverse transcriptase inhibitors (N(t)RTIs), or administered as the D/C/F/TAF STR, as a recommended treatment option,^2^ or recommended in certain clinical situations, such as for those patients who may have uncertain adherence, those who require a regimen with a high genetic barrier to resistance, or those patients who may not have resistance results available.^1,3^

We report HIV-1 resistance data in participants with protocol-defined virologic failure (PDVF) in AMBER and in EMERALD (participants with PDVR). In addition, HIV-1 proviral DNA from baseline samples (VL <50 copies/mL) was retrospectively analyzed to assess the prevalence of archived RAMs in participants with prior VF or PDVR, who were most likely to have baseline resistance, in EMERALD.

## Methods

### AMBER and EMERALD study designs and participants

Detailed methods for the international, multicenter, Phase III, randomized, active-controlled noninferiority trials, AMBER (TMC114FD2HTX3001; NCT02431247)^10^ and EMERALD (TMC114IFD3013; NCT02269917),^11^ have been reported previously.

AMBER (double-blind) included ART-naive adults with a screening plasma VL ≥1,000 copies/mL, CD4^+^ cell count >50 cells/mm^3^, and HIV-1 virus with genotypic sensitivity to DRV, emtricitabine, and tenofovir. EMERALD (open-label) included ART-experienced, virologically suppressed adults (VL <50 copies/mL for ≥2 months before screening; one 50 ≤ VL <200 copies/mL within 12 months before screening allowed) while on a stable bPI regimen (DRV/ritonavir or DRV/cobicistat once daily, atazanavir/ritonavir or atazanavir/cobicistat once daily, or lopinavir/ritonavir twice daily) + F/TDF regimens for ≥6 months. Patients with prior experience with multiple antiretrovirals and prior VF were eligible for study participation, provided they had no previous VF on DRV-based regimens and absence of DRV RAMs,^14^ if historical genotypes were available. No restriction was placed on the presence of emtricitabine or tenofovir RAMs or any other RAMs.

The primary outcome of both studies was to demonstrate noninferior efficacy of D/C/F/TAF 800/150/200/10 mg once daily compared with the control arm (bPI + F/TDF 200/300 mg fixed-dose combination [FDC] in EMERALD or D/C 800/150 mg FDC + F/TDF 200/300 mg FDC in AMBER) at week 48. Patients continued receiving D/C/F/TAF beyond week 48, provided they consented and continued to derive benefit as assessed by the investigator. Primary outcome measures were the proportion of participants with VL <50 copies/mL (virologic response rate) by the FDA-snapshot analysis at week 48 (AMBER) or PDVR (confirmed VL ≥50 copies/mL or premature discontinuations, irrespective of reason with last VL ≥50 copies/mL) cumulative through week 48 (EMERALD). The FDA-snapshot virologic response and VF rate (VL ≥50 copies/mL) were also assessed as efficacy endpoints in EMERALD.

### Virology assessments

In both studies, plasma HIV-1 RNA was quantified at screening, baseline, weeks 2, 4, 8, and 12, and every 12 weeks thereafter with the COBAS^®^ AmpliPrep/COBAS^®^ TaqMan HIV-1 Test V2.0 (Roche Diagnostics, Basel, Switzerland). Standard resistance testing was done when HIV-1 RNA was ≥400 copies/mL.

#### ART-naive participants (AMBER)

Genotypic resistance testing was performed using GenoSure^®^ MG (HIV-1 protease [PR]/reverse transcriptase [RT] genotype assay; Monogram Biosciences, South San Francisco, CA) at screening. Genotypic and phenotypic testing was performed using PhenoSense^®^ GT (combined HIV-1 PR/RT genotype/phenotype) postbaseline in participants with PDVF with VL ≥400 copies/mL at failure (preferably confirmed, or at unconfirmed) or at later time points.^10^ PDVF was defined as virologic nonresponse (VL <1 log_10_ reduction from baseline and ≥50 copies/mL at week 8, confirmed at next visit) or virologic rebound (confirmed VL ≥50 copies/mL after confirmed, consecutive VL <50 copies/mL or confirmed VL >1 log_10_ increase from the nadir) and/or viremia at the final time point (VL ≥400 copies/mL at study endpoint or study discontinuation after week 8).

Deep sequencing using NGS GenoSure MG (Illumina MiSeq; codon variants >1%) was performed retrospectively on samples from participants with HIV-1 viruses that showed emerging resistance-associated mutations.

#### Virologically suppressed ART-experienced participants (EMERALD)

Postbaseline HIV-1 PR and RT genotypic resistance testing was performed using GenoSure MG for participants with PDVR (confirmed VL ≥50 copies/mL or single unconfirmed last on-treatment HIV-1 RNA ≥50 copies/mL at premature discontinuation) and who had a VL ≥400 copies/mL at failure (preferably confirmed, or at unconfirmed) or at later time points, including participants who discontinued with a last single VL ≥400 copies/mL.

EMERALD included virologically suppressed adults, and therefore no standard resistance testing could be performed on screening or baseline samples. HIV-1 proviral DNA was analyzed retrospectively using GenoSure Archive^®^ on baseline samples (VL <50 copies/mL) from participants with PDVR or prior VF (i.e., the participants most likely to have HIV-1 viruses with baseline resistance).

### Genotypic analysis

Evaluation of screening and treatment-emergent RAMs (AMBER), or the presence of on-treatment (EMERALD) RAMs, was based on the PI, N(t)RTI, NNRTI, and integrase inhibitor International Antiviral Society [IAS]-USA mutation list.^14^ RAMs were considered developing (emerging) if they were detected postbaseline but not at screening.

### Phenotypic analysis

Assessments of antiretroviral drug susceptibility (predicted phenotype, “sensitive,” “resistance possible,” or “resistant”) were based on the genotype and applied algorithms (GenoSure MG or GenoSure Archive) or on the true phenotype, expressed as the fold change (FC) in the 50% effective concentration in cell-based assays (EC_50_) below/equal or above, respectively, the lower clinical cutoff (when available) or biological cutoff (otherwise) for the PhenoSense GT report.

## Results

### ART-naive participants (AMBER)

#### Baseline characteristics

Overall, 18% of participants had HIV-1 VL ≥100,000 copies/mL, and median baseline CD4^+^ count was 453 cells/mm.^3,10^ A majority of participants were infected with HIV-1 subtype B (71%).

As depicted by the protocol at screening, all enrolled participants had HIV-1 viruses sensitive to DRV, emtricitabine, and tenofovir based on the genotype report. As expected in a treatment-naive population, few participants had viruses with ≥1 primary PI RAMs (2%) or ≥1 DRV RAMs (1%) (Table 1). No participants had HIV-1 with ≥3 DRV RAMs, which is correlated with DRV resistance.^15^ Secondary PI RAMs were observed in HIV-1 of almost all participants (98%), reflecting the polymorphic nature of these mutations. NNRTI and N(t)RTI RAMs were detected in HIV-1 of 16% and 5% of participants, respectively, with K103N and polymorphic E138E/A mutation being the most prevalent NNRTI RAMs (both 4%) and A62A/V (3%) the most prevalent N(t)RTI RAM (Table 1). No RAMs related to emtricitabine or tenofovir were detected.

**Table 1.** Amber Genotype at Screening

	D/C/F/TAF 800/150/200/10 mg* N* = 362	Control regimen (D/C + F/TDF)* N* = 363	Total* N* = 725
Participants with genotype^a^ at screening	*N* = 361^b^	*N* = 362^b^	*N* = 723
≥1 DRV RAM^c^, *n* (%)	3 (1)	4 (1)	7 (1)
V11V/A/I/T	3 (1)	3 (1)	6 (1)
L33F	0	1 (<1)	1 (<1)
≥1 primary PI RAM^c^, *n* (%)	7 (2)	8 (2)	15 (2)
M46M/I/L	0	3 (1)	3 (<1)
Q58Q/E	4 (1)	4 (1)	8 (1)
V82L	1 (<1)	0	1 (<1)
L90M	2 (1)	1 (<1)	3 (<1)
≥1 secondary PI RAM, *n* (%)	354 (98)	354 (98)	708 (98)
≥1 N(t)RTI RAM^c^, *n* (%)	18 (5)	16 (4)	34 (5)
M41M/L	3 (1)	2 (1)	5 (1)
A62A/V	10 (3)	11 (3)	21 (3)
D67N	2 (1)	1 (<1)	3 (<1)
K70K/R	0	1 (<1)	1 (<1)
V75I	1 (<1)	0	1 (<1)
L210W	2 (1)	1 (<1)	3 (<1)
K219Q	2 (1)	1 (<1)	3 (<1)
≥1 NNRTI RAM^c^, *n* (%)	55 (15)	63 (17)	118 (16)
V90I	9 (2)	9 (2)	18 (2)
K103N	12 (3)	14 (4)	26 (4)
V106I	7 (2)	11 (3)	18 (2)
E138E/A	12 (3)	16 (4)	28 (4)
V179D	4 (1)	2 (1)	6 (1)

^a^ GenoSure^®^ MG.

^b^ One participant in each arm had failed screening genotypes and were enrolled based on local genotypes.

^c^ IAS-USA mutations^16^; all observed single and mixture of mutations were concatenated by RAM position.

D/C/F/TAF, darunavir/cobicistat/emtricitabine/tenofovir alafenamide once daily; Control regimen (D/C + F/TDF), darunavir/cobicistat plus emtricitabine/tenofovir disoproxil fumarate once daily; DRV, darunavir; RAM, resistance-associated mutation; PI, protease inhibitor; N(t)RTI, nucleos(t)ide analog reverse transcriptase inhibitor; NNRTI, non-nucleoside analog reverse transcriptase inhibitor.

Subgroup analyses showed that there was no effect of HIV-1 subtype (B, non-B), presence of baseline primary PI and/or DRV RAMs, or N(t)RTI RAMs, NNRTI RAMs, M184I/V, and number of primary PI RAMs on virologic response at week 48 (FDA snapshot) (data not shown). Of the seven participants with viruses harboring a DRV RAM at screening (three in the D/C/F/TAF arm and four in the control arm), six had virologic response at week 48 and one discontinued due to other reasons with last available VL <50 copies/mL (FDA snapshot).

#### Postbaseline resistance

Through 48 weeks, 8 (D/C/F/TAF) and 6 (control) participants had PDVF, with virologic rebound occurring most frequently (Supplementary Fig. S1). Paired screening and postbaseline on-treatment genotypes were available for seven and two participants, respectively (Table 2).

**Table 2.** Amber and Emerald: Postbaseline Resistance Through Week 48

Study	ART	Participants, N	Participants with PDVF, *n* (%)	PDVF participants with postbaseline genotype data, *n* (%)	Non-PDVF participants with postbaseline genotype data, *n* (%)	Participants with ≥1 RAM^a^ postbaseline^b^, n (%)	
						Reverse transcriptase	Protease
						FTC or TFV	Primary PI or DRV
AMBER	**D/C/F/TAF**	**362**	**8 (2)**	**7 (2)**	**3 (1)**	**M184I/V^c^; *n* = 1**	**0**
	Control regimen (D/C + F/TDF)	363	6 (2)	2 (1)	2 (1)	0	0
EMERALD	**D/C/F/TAF**	**763**	**19 (2)**	**1^e^ (<1)**	**2^f^ (<1)**	**0**	**0**
	Control regimen (bPI^d^ + F/TDF)	378	8 (2)	3^g^ (1)	0	0	0
Total	**D/C/F/TAF**	**1125**	**27 (2)**	**8 (1)**	**5 (<1)**	**1 (<1)**	**0**

All data from the DCFTAF groups are bolded.

^a^ IAS-USA 2015 mutation list^16^; all observed single and mixture of mutations were concatenated by RAM position.

^b^ At unconfirmed or confirmed virologic failure or later time point(s).

^c^ Conferring phenotypic resistance to emtricitabine and lamivudine, but retaining sensitivity to abacavir, tenofovir, stavudine, and zidovudine; this participant had HIV-1 virus with transmitted efavirenz and nevirapine resistance shown by the presence of K103N at screening; MI84V was detected by deep sequencing (9%) at screening.

^d^ At screening, 266 participants were on boosted DRV (*n* = 202 DRV/ritonavir; *n* = 64 DRV/cobicistat), 82 on boosted atazanavir (*n* = 81 atazanavir/ritonavir; *n* = 1 atazanavir/cobicistat), and 30 on lopinavir/ritonavir.

^e^ One rebounder in the D/C/F/TAF arm had an N(t)RTI thymidine analog-associated mutation (TAM), D67D/N.

^f^ One of these participants had a K103K/N NNRTI RAM conferring resistance to efavirenz and nevirapine.

^g^ One rebounder in the control arm had an NNRTI RAM, E138E/G, conferring resistance to rilpivirine; this mutation was not related to any of the study drugs and was probably related to previous ART use.

ART, antiretroviral treatment; PDVF, protocol-defined virologic failure; RAM, resistance-associated mutation; FTC, emtricitabine; TFV, tenofovir; bPI, boosted protease inhibitor; DRV, darunavir; D/C/F/TAF, darunavir/cobicistat/emtricitabine/tenofovir alafenamide; F/TDF, emtricitabine/tenofovir disoproxil fumarate.

No DRV, primary PI, or tenofovir RAMs emerged in HIV-1 of any participant (Table 2).

The N(t)RTI RAM M184I/V, conferring phenotypic resistance to emtricitabine (FC in EC_50_ = 56) and lamivudine (FC = 143), but retaining sensitivity to abacavir, stavudine, tenofovir, and zidovudine, was identified at week 36 post-PDVF by Sanger sequencing in only one participant receiving D/C/F/TAF. M184V was detected pretreatment at screening by deep sequencing as a minority variant (9%). At week 12 only the wild-type genotype was detected, and at week 36, M184I (32%) and M184V (67%) were detected.

This participant had HIV-1 virus with transmitted NNRTI (efavirenz [FC = 7]/nevirapine [FC = 39]) resistance due to the presence of K103N at screening. This participant was discontinued from the study due to noncompliance after the week 48 database lock. Further testing showed that the observed DRV plasma concentrations for this participant were low (ranging from 32.0 to 192 ng/mL, except at week 4 [1,440 ng/mL]) and much lower than the anticipated DRV steady-state predose plasma concentration for this participant (∼692 ng/mL), which could be an indication that the participant did not take the medication regularly as recommended.

All other participants had virus that remained susceptible to all drugs in the treatment regimens based on genotypic/phenotypic assessments (PhenoSense GT assay).

One participant in the D/C/F/TAF arm had HIV-1 that developed the secondary PI RAM I62I/V. Three participants in the D/C/F/TAF arm and two participants in the control arm, none of whom met the definition of PDVF, had postbaseline off-treatment genotype data just after discontinuation (Table 2). One of the three participants in the D/C/F/TAF arm had virus with an emerging secondary PI RAM I64I/L/M. As expected, these secondary PI RAMs had no effect on PI susceptibility.

### Virologically suppressed ART-experienced participants (EMERALD)

#### Baseline characteristics

Most participants received DRV/ritonavir or DRV/cobicistat at screening. Median time since diagnosis was 9.26 years (interquartile range 4.22–18.12 years).^11^

Of 1,141 participants, 664 (58%) had received ≥5 previous antiretrovirals (including screening ART and PI booster counted as a separate antiretroviral) and 312 (27%) had received ≥8 previous antiretrovirals; 472 (41%) had received ≥2 PIs, 474 (42%) ≥3 N(t)RTIs, 340 (30%) ≥1 NNRTI, and 63 (6%) ≥1 integrase inhibitor.

In total, 169 (15%) participants had previous antiretroviral VF (80 [7%] participants on a PI, 130 [11%] on an N(t)RTI, 74 [6%] on an NNRTI, and 10 [1%] on an integrase inhibitor).

One participant who had HIV-1 with a V11I DRV RAM, which was a protocol deviation, reported on a historical genotype, was randomized and had a virologic response throughout the study until week 36, after which the participant was lost to follow-up.

#### Archived baseline resistance

HIV-1 proviral DNA from baseline samples from all participants with prior VF (*N* = 169) or with PDVR (*N* = 27) (i.e., the subgroup most likely to have baseline resistance) was analyzed.

In the subgroup of all EMERALD participants with previous VF and genoarchive data (*N* = 140; 98 D/C/F/TAF and 42 bPI + F/TDF), 4% (*n* = 6; 4 D/C/F/TAF and 2 bPI + F/TDF) had viruses with DRV RAMs, 4% with tenofovir RAMs (*n* = 5) (Table 3), and 21% with ≥3 thymidine analog-associated mutations (TAMs) (*n* = 29). Emtricitabine RAMs were seen in 38% (*n* = 53), mainly at RT position 184 (35%) (Table 3). Predicted phenotype showed that 100% of participants had HIV-1 susceptible to DRV, 59% to emtricitabine, and 76% to tenofovir; 83% to all PIs, 51% to all N(t)RTIs, and 61% to all NNRTIs (Table 3). All these participants with virus harboring archived DRV, emtricitabine, and tenofovir resistance achieved virologic response (FDA snapshot) at week 48 or at last on-treatment VL.

**Table 3.** Emerald: Prevalence of Baseline RAMS in HIV-1 Proviral DNA for Participants with Previous Virologic Failure

	D/C/F/TAF 800/150/200/10 mg* N* = 763	Control regimen (bPI + F/TDF)* N* = 378	Total* N* = 1,141
Participants with previous VF, *N*	116	53	169
Participants with previous VF and genoarchive^a^ data at baseline, *N*^b^	98	42	140
Genotypic Susceptibility, *n* (%):			
All PIs	80 (82)	36 (86)	116 (83)
Boosted ATV	91 (93)	40 (95)	131 (94)
Boosted DRV	98 (100)	42 (100)	140 (100)
Boosted LPV	94 (96)	40 (95)	134 (96)
All N(t)RTIs	53 (54)	19 (45)	72 (51)
FTC	62 (63)	21 (50)	83 (59)
TFV	76 (78)	30 (71)	106 (76)
All NNRTIs	56 (57)	29 (69)	85 (61)
All INIs	86 (88)	39 (93)	125 (89)
DTG	97 (99)^c^	41 (98)^c^	138 (99)
EVG	89 (91)	40 (95)	129 (92)
RTG	86 (88)	39 (93)	125 (89)
≥1 DRV RAMs^d^, *n* (%)	4^e^ (4)	2 (5)	6 (4)
L33L/F	0	1 (2)	1 (1)
T74T/P	0	1 (2)	1 (1)
L76L/V	1 (1)	0	1 (1)
I84I/V	4 (4)	0	4 (3)
≥1 primary PI RAMs^d^, *n* (%)	20 (20)	7 (17)	27 (19)
D30D/N	4 (4)	0	4 (3)
M46M/I/L	8 (8)	5 (12)	13 (9)
I50I/L	0	1 (2)	1 (1)
Q58Q/E	0	1 (2)	1 (1)
T74T/P	0	1 (2)	1 (1)
L76L/V	1 (1)	0	1 (1)
V82V/A/T	1 (1)	2 (5)	3 (2)
I84I/V	4 (4)	0	4 (3)
N88N/S	2 (2)	0	2 (1)
L90L/I/M	8 (8)	3 (7)	11 (8)
≥1 N(t)RTI RAMs, *n* (%)	46 (47)	23 (55)	69 (49)
≥1 tenofovir RAMs^d^, *n* (%)	4 (4)	1 (2)	5 (4)
K65K/R	4 (4)	0	4 (3)
K70K/D/E/N	0	1 (2)	1 (1)
≥1 TAMs^d,f^*n* (%)	34 (35)	14 (33)	48 (34)
M41M/L	18 (18)	8 (19)	26 (19)
D67D/N	10 (10)	7 (17)	17 (12)
K70K/R	18 (18)	6 (14)	24 (17)
L210L/W	9 (9)	3 (7)	12 (9)
T215A/C/D/E/F/G/I/L/N/S/T/Y	20 (20)	12 (29)	32 (23)
K219K/E/Q	11 (11)	4 (10)	15 (11)
≥1 emtricitabine RAMs^d^, *n* (%)	35 (36)	18 (43)	53 (38)
K65K/R	4 (4)	0	4 (3)
M184M/I/V	31 (32)	18 (43)	49 (35)
≥1 NNRTI RAMs, *n* (%)	44 (45)	14 (33)	58 (41)
V90V/I	5 (5)	3 (7)	8 (6)
A98A/G/S	5 (5)	1 (2)	6 (4)
L100L/I	2 (2)	0	2 (1)
K101K/E/P/Q/T	7 (7)	1 (2)	8 (6)
K103K/N/R/S	19 (19)	6 (14)	25 (18)
V106V/A/I	3 (3)	1 (2)	4 (3)
V108V/I/M	6 (6)	1 (2)	7 (5)
E138E/A/G/K/P/Q/R	13 (13)	1 (2)	14 (10)
V179V/A/F/I/T	2 (2)	0	2 (1)
Y181Y/C	8 (8)	3 (7)	11 (8)
Y188Y/H/L	3 (3)	0	3 (2)
G190G/A/R/S	8 (8)	4 (10)	12 (9)
H221H/Y	1 (1)	1 (2)	2 (1)
P225P/H	1 (1)	1 (2)	2 (1)
M230M/L	0	1 (2)	1 (1)
≥1 Primary INI RAMs, *n* (%)	5 (5)	1 (2)	6 (4)
T66T/I	2 (2)	0	2 (1)
Q148Q/H/R	1 (1)	1 (2)	2 (1)
N155N/H	2 (2)	0	2 (1)

^a^ GenoSure Archive^®^.

^b^ Denominator for the prevalence of baseline RAMs.^16^

^c^ Two participants showed possible resistance to DTG and full resistance to RTG and EVG (integrase inhibitor RAMs: G140S, Q148H and G140A, Q148R) on the genotype report. Both participants had previously virologically failed on RTG.

^d^ Observed single and mixture of mutations were concatenated by RAM position.

^e^ In one participant, 2 DRV RAMs were observed (I84I/V and L76L/V).

^f^ Thymidine analog-associated mutations (TAMs): M41L, D67N, K70R, L210W, T215Y/F, K291Q/E.^16,17^

Tenofovir resistance is associated with the presence of ≥3 TAMs, inclusive of either M41L or L210W.

D/C/F/TAF, darunavir/cobicistat/emtricitabine/tenofovir alafenamide once daily; Control regimen (bPI + F/TDF), boosted protease inhibitor plus emtricitabine/tenofovir disoproxil fumarate once daily (At screening, 266 participants were on boosted DRV [*n* = 202 DRV/ritonavir; *n* = 64 DRV/cobicistat], 82 on boosted ATV [*n* = 81 ATV/ritonavir; *n* = 1 ATV/cobicistat], and 30 on LPV/ritonavir)

VF, virologic failure; PI, protease inhibitor; ATV, atazanavir; DRV, darunavir; LPV, lopinavir; N(t)RTI, nucleos(t)ide analog reverse transcriptase inhibitor; FTC, emtricitabine; TFV, tenofovir; NNRTI, non-nucleoside analogue reverse transcriptase inhibitor; INI, integrase inhibitor; DTG, dolutegravir; EVG, elvitegravir; RTG, Raltegravir; RAM, resistance-associated mutation.

In the subgroup of participants with PDVR and genoarchive data (*n* = 24), none had HIV-1 with archived RAMs to DRV, emtricitabine, or tenofovir (Table 4). Also no primary PI RAMs nor TAMs were observed.

**Table 4.** Emerald: Prevalence of Baseline RAMS in HIV-1 Proviral DNA for Participants with PDVR

	D/C/F/TAF 800/150/200/10 mg* N* = 763	Control regimen (bPI + F/TDF)* N* = 378	Total* N* = 1,141
Participants with PDVR, *N*	19	8	27
Participants with PDVR and genoarchive^a^ data at baseline, *N*^b^	17	7	24
Genotypic Susceptibility, *n* (%)			
All PIs	16^c^ (94)	7 (100)	23^c^ (96)
All N(t)RTIs	17 (100)	7 (100)	24 (100)
All NNRTIs	13 (76)	7 (100)	20 (83)
All INIs	17 (100)	7 (100)	24 (100)
≥1 DRV RAMs/primary PI RAMs, *n* (%)	0	0	0
≥1 tenofovir RAMs, *n* (%)	0	0	0
≥1 TAMs *n* (%)	0	0	0
≥1 emtricitabine RAMs, *n* (%)	0	0	0

^a^ GenoSure Archive.

^b^ Denominator for the prevalence of baseline RAMs.^16^

^c^ One participant had HIV-1 virus not susceptible to nelfinavir or unboosted atazanavir.

D/C/F/TAF, darunavir/cobicistat/emtricitabine/tenofovir alafenamide once daily; Control regimen (bPI + F/TDF), boosted protease inhibitor plus emtricitabine/tenofovir disoproxil fumarate once daily (At screening, 266 participants were on boosted DRV [*n* = 202 DRV/ritonavir; *n* = 64 DRV/cobicistat], 82 on boosted atazanavir [*n* = 81 atazanavir/ritonavir; *n* = 1 atazanavir/cobicistat], and 30 on lopinavir/ritonavir); PDVR, protocol-defined virologic rebound; PI, protease inhibitor; N(t)RTI, nucleos(t)ide analog reverse transcriptase inhibitor; NNRTI, non-nucleoside analogue reverse transcriptase inhibitor; INI, integrase inhibitor; DRV, darunavir; RAM, resistance-associated mutation; TAM, thymidine analog-associated mutation.

#### Postbaseline resistance

In EMERALD, as there were few participants with PDVR throughout 48 weeks (19/763 D/C/F/TAF arm and 8/378 control arm), few samples were eligible for postbaseline genotyping (participants whose VL rebounded with VL ≥400 copies/mL at failure or at later time points or at discontinuation). Only three participants in the D/C/F/TAF arm and zero participants in the control arm had virologic rebound using the VL ≥200 copies/mL threshold. One participant whose VL rebounded in the D/C/F/TAF arm and three participants that rebounded in the control arm had postbaseline genotypes (Table 2 and Supplementary Fig. S2).

For the four postbaseline PR and RT sequences, the predicted phenotypic assessment (based on the GenoSure MG assay) was that all participants had HIV-1 virus susceptible to all the drugs in the treatment regimen and no DRV, primary PI, tenofovir, or emtricitabine RAMs were observed (Table 2).

One participant with PDVR in the D/C/F/TAF arm had virus with one TAM, D67D/N, and one participant with PDVR in the control arm had HIV-1 with an NNRTI RAM, E138E/G, conferring resistance to rilpivirine. Two further participants in the D/C/F/TAF arm who discontinued at week 12, but whose VL did not rebound (assessed as “no VL data in the week 48 window” by the FDA-snapshot analysis), had a genotype at a posttreatment follow-up visit. One of these participants had virus with a K103K/N NNRTI RAM conferring resistance to efavirenz, which was probably related to previous use of efavirenz, emtricitabine, and TDF (Atripla).

## Discussion

The week 48 analyses of the two investigational Phase III, randomized, controlled trials showed that the D/C/F/TAF once-daily STR had a response rate of 91% in ART-naive participants in AMBER and 95% in virologically suppressed participants in EMERALD, with VF rates of 4% and 1%, respectively, and a high genetic barrier to resistance. Through week 48 in both studies, no treatment-emergent DRV or primary PI RAMs were observed in the HIV-1 of 1,125 participants receiving D/C/F/TAF, or in 629 participants receiving boosted DRV in combination with F/TDF in the control arms. No tenofovir RAM or tenofovir resistance was observed in either trial. The most common N(t)RTI RAM, M184I/V (conferring high-level *in vitro* resistance to emtricitabine and lamivudine), was identified post-VF in HIV-1 of only one participant in the D/C/F/TAF arm of AMBER. However, the presence of M184I/V is not a contraindication to the use of emtricitabine and lamivudine, because these RAMs increase tenofovir susceptibility and decrease viral replication fitness.^18^ For this participant, M184V was detected in the virus pretreatment by deep sequencing (Illumina MiSeq) as a minority variant (9%). This participant also had HIV-1 with transmitted NNRTI (efavirenz/nevirapine) resistance as shown by the presence of K103N at screening. This participant was potentially nonadherent as indicated by the low DRV plasma concentrations, which were lower than the steady-state predose concentration, and the participant discontinued from the study due to noncompliance.

These results are consistent with those of a previous meta-analysis of postbaseline resistance among participants in the DRV 800 mg once-daily dosing arms from seven clinical Phase II and III studies.^9^ Only four of 1,686 (0.2%) participants had viruses that developed postbaseline primary PI and/or DRV RAMs, and only one of 1,686 (<0.1%) participant had HIV-1 that lost DRV phenotypic susceptibility after having previously failed lopinavir/ritonavir. To our knowledge, no participant that has not previously failed another PI has ever failed with HIV-1 that was phenotypically resistant to DRV. Only 10 of 1,103 participants from the seven clinical trials using an N(t)RTI backbone (0.9%) had viruses that developed ≥1 N(t)RTI RAM, and eight of those had HIV-1 harboring the emtricitabine RAM M184I/V; no tenofovir RAM or tenofovir resistance was detected. Other studies investigating F/TAF containing STR regimens showed similar results. Two Phase III studies investigating virologically suppressed participants who switched to rilpivirine/emtricitabine/tenofovir alafenamide reported zero and two M184V RAMs postbaseline out of 438^19^ and 316^20^ participants, respectively. Of 959 virologically suppressed participants assigned to the elvitegravir/cobicistat/emtricitabine/tenofovir (E/C/F/TAF) alafenamide arm, one had HIV-1 that developed M184M/I.^21^ In two Phase III studies in ART-naive adults investigating the E/C/F/TAF STR (in total 866 participants were in the E/C/F/TAF arm), development of the M184V/I RAM was observed in HIV-1 of 11 E/C/F/TAF participants and the K65R/N RAM in viruses of two participants.^22,23^ In Phase III studies of bictegravir, emtricitabine, and tenofovir alafenamide (B/F/TAF) in ART-naive ^24,25^ and virologically suppressed adults,^26,27^ no treatment emergent resistance to the components of the treatment regimen were identified.

In AMBER through week 48, only eight out of 362 ART-naive participants in the D/C/F/TAF arm and six out of 363 in the control arm had PDVF, with paired screening and postbaseline on-treatment genotypes available for seven and two participants, respectively.^10^ Similarly, as there were few participants whose VL rebounded through 48 weeks in EMERALD, with most participants having low VL values, samples from only one participant who rebounded in the D/C/F/TAF arm, and three who rebounded in the control arm were eligible for post-week 48 HIV-1 genotypic resistance analysis. Most participants with rebound had resuppressed VL by week 48 and confirmed rebounds ≥200 copies/mL occurred in only three participants in the D/C/F/TAF arm and none in the control arm.^11^

In EMERALD, exclusion criteria were less restrictive (and thus participants may be more representative of the real world) than in other recent switch studies for HIV ART.^26–33^ While participants in EMERALD were ART experienced, with 58% of participants having received ≥5 previous antiretroviral agents, and 15% had previous VF, few participants had PDVR cumulative through week 48. Subgroup analyses by previous antiretroviral use and VF showed no effect on rebound rates, virologic response, and VF rates by the FDA-snapshot analysis.^11,34^ There was no exclusion on the basis of tenofovir or emtricitabine RAMs. In EMERALD D/C/F/TAF participants with previous VF (*n* = 98) at baseline, 37% (*n* = 36) had HIV-1 not fully susceptible to emtricitabine and 22% (*n* = 22) not fully susceptible to tenofovir (based on the genoarchive assay); baseline archived RAMs to tenofovir (4 D/C/F/TAF participants) and emtricitabine (35 D/C/F/TAF participants, mainly 31 with M184I/V) were observed. Nineteen percent (*n* = 19) had HIV-1 with ≥3 TAMs, of which 9% (*n* = 9) had virus with L210W and 18% (*n* = 18) with M41L (tenofovir resistance is also associated with the presence of ≥3 TAMs, inclusive of either M41L or L210W).^17^ This observed archived resistance did not preclude virologic response in D/C/F/TAF participants. In participants with PDVR, none had viruses with archived RAMs to DRV, emtricitabine, and tenofovir. The clinical utility of proviral DNA resistance has yet to be fully established. These next-generation sequencing genotypic assays that analyze HIV-1 proviral DNA might miss some previous drug resistance mutations found in plasma viral RNA and vice versa, and they should be interpreted with caution.^1,35,36^

In EMERALD, the only exclusion criterion for presence of mutations was the presence of ≥1 DRV RAMs if historical genotypes were available (or if not, previous VF on DRV-based regimens). This exclusion criterion was introduced in line with the DRV once-daily indication in ART-experienced virologically failing participants. Efficacy from a pooled dataset of studies in highly ART-experienced participants, who had received treatment with DRV/ritonavir 600/100 mg twice daily, was shown to be compromised only in the presence of ≥3 DRV RAMs in the background of a high number of PI RAMs (median of 14 or 15, respectively).^15^ There were three participants with HIV-1 harboring a DRV RAM (V11I) pre-D/C/F/TAF treatment in AMBER, all who had virologic response at week 48 (or at discontinuation) by the FDA-snapshot analysis. These observations are also in line with Lathouwers *et al.*^9^ who showed that of 14 participants who had viruses with a DRV RAM at baseline and received DRV 800 mg once daily-based regimens, all achieved virologic response. In EMERALD, in the subgroup with previous VF or PDVR receiving D/C/F/TAF with genoarchive data, 4 (all previous VFs) participants had HIV-1 with archived DRV RAMs (all had viruses harboring one DRV RAM and one participant had virus with 2 DRV RAMs) at baseline. All of these 4 participants had virologic response at week 48 (FDA-snapshot analysis).

D/C/F/TAF may have an important role for treating patients with uncertain adherence or for rapid initiation in patients for whom resistance testing results are not yet available.^1,3^ Furthermore, D/C/F/TAF does not require HLA B*5701 screening or hepatitis testing before treatment initiation. All these characteristics suggest D/C/F/TAF is a feasible option in a test and treat setting or for ART-naive patients, where rapid treatment is justified. D/C/F/TAF is being evaluated in the first known Phase III trial (single arm; *N* = 109) of an STR in a rapid initiation model in the DIAMOND study (NCT03227861).^37^ In the primary intent-to-treat analysis, 84% of patients (92/109) achieved virologic response at week 48 (FDA-snapshot analysis).

In conclusion, D/C/F/TAF has a high genetic barrier to resistance in ART-naive and ART-experienced adults living with HIV-1, even in those with virus harboring archived study drug RAMs at baseline. The D/C/F/TAF STR combines the efficacy and high genetic barrier to resistance of DRV with the safety advantages of TAF to provide a new option for people living with HIV-1.

## Supplementary Material

Supplemental data

Supplemental data

## AMBER Principal Investigators

**Belgium:** S De Wit, E Florence, L Vandekerckhove, B Vandercam; **Canada:** J Brunetta, M Klein, D Murphy, A Rachlis, S Walmsley; **France:** F Ajana, L Cotte, P-M Girard, C Katlama, J-M Molina, I Poizot-Martin, F Raffi, D Rey, J Reynes, E Teicher, and Y Yazdanpanah; **Germany:** K Arastéh, M Bickel, J Bogner, S Esser, G Faetkenheuer, H Jessen, W Kern, J Rockstroh, C Spinner, H-J Stellbrink, and A Stoehr; **Italy:** A Antinori, F Castelli, A Chirianni, A De Luca, A Di Biagio, M Galli, A Lazzarin, F Maggiolo, R Maserati, and C Mussini; **Poland:** A Garlicki, J Gasiorowski, W Halota, A Horban, M Parczewski, and A Piekarska; **Russia:** E Belonosova, O Chernova, N Dushkina, V Kulagin, E Ryamova, A Shuldyakov, N Sizova, O Tsybakova, E Voronin, and A Yakovlev; **Spain:** A Antela, JR Arribas, J Berenguer, J Casado, V Estrada, MJ Galindo, M Garcia Del Toro, JM Gatell, M Gorgolas, F Gutierrez, MDM Gutierrez, E Negredo, JA Pineda, D Podzamczer, J Portilla Sogorb, A Rivero, R Rubio, P Viciana, and I De Los Santos; **UK:** A Clarke, BG Gazzard, MA Johnson, C Orkin, I Reeves, and L Waters; **USA:** P Benson, L Bhatti, F Bredeek, G Crofoot, D Cunningham, E DeJesus, J Eron, F Felizarta, R Franco, J Gallant, D Hagins, K Henry, D Jayaweera, C Lucasti, C Martorell, C McDonald, J McGowan, A Mills, J Morales-Ramirez, D Prelutsky, M Ramgopal, B Rashbaum, P Ruane, J Slim, A Wilkin, and J deVente.

## EMERALD Principal Investigators

**Belgium**: S De Wit, E Florence, M Moutschen, E Van Wijngaerden, L Vandekerckhove, and B Vandercam; **Canada**: J Brunetta, B Conway, M Klein, D Murphy, A Rachlis, S Shafran, and S Walmsley; **France**: F Ajana, L Cotte, P-M Girard, C Katlama, J-M Molina, I Poizot-Martin, F Raffi, D Rey, J Reynes, E Teicher, and Y Yazdanpanah; **Poland**: J Gasiorowski, W Halota, A Horban, A Piekarska, and A Witor; **Spain**: JR Arribas, I Perez-Valero, J Berenguer, J Casado, JM Gatell, F Gutierrez, MJ Galindo, MDM Gutierrez, JA Iribarren, H Knobel, E Negredo, JA Pineda, D Podzamczer, J Portilla Sogorb, F Pulido, C Ricart, A Rivero, I Santos Gil; **Sweden**: A Blaxhult, L Flamholc, M Gisslèn, and A Thalme; **Switzerland**: J Fehr, A Rauch, and M Stoeckle; **UK**: A Clarke, BG Gazzard, MA Johnson, C Orkin, F Post, A Ustianowski, and L Waters; **USA**: J Bailey, P Benson, L Bhatti, I Brar, UF Bredeek, C Brinson, G Crofoot, D Cunningham, E DeJesus, C Dietz, R Dretler, J Eron, F Felizarta, C Fichtenbaum, J Gallant, J Gathe, D Hagins, S Henn, WK Henry, G Huhn, M Jain, C Lucasti, C Martorell, C McDonald, A Mills, J Morales-Ramirez, K Mounzer, R Nahass, H Olivet, O Osiyemi, D Prelutsky, M Ramgopal, B Rashbaum, G Richmond, P Ruane, A Scarsella, A Scribner, P Shalit, D Shamblaw, J Slim, K Tashima, G Voskuhl, D Ward, A Wilkin, and J de Vente.
