# Supplementary material for: Week 48 Resistance Analyses of the Once-Daily, Single-Tablet Regimen Darunavir/Cobicistat/Emtricitabine/Tenofovir Alafenamide (D/C/F/TAF) in Adults Living with HIV-1 from the Phase III Randomized AMBER and EMERALD Trials
Source: AIDS Res Hum Retroviruses. 2019 Dec 31;36(1):48–57. doi: 10.1089/aid.2019.0111 (PMC6944133; doi:10.1089/aid.2019.0111)
Supplement: Supplemental data [file Supp_Fig1.pdf]

Supplementary Data

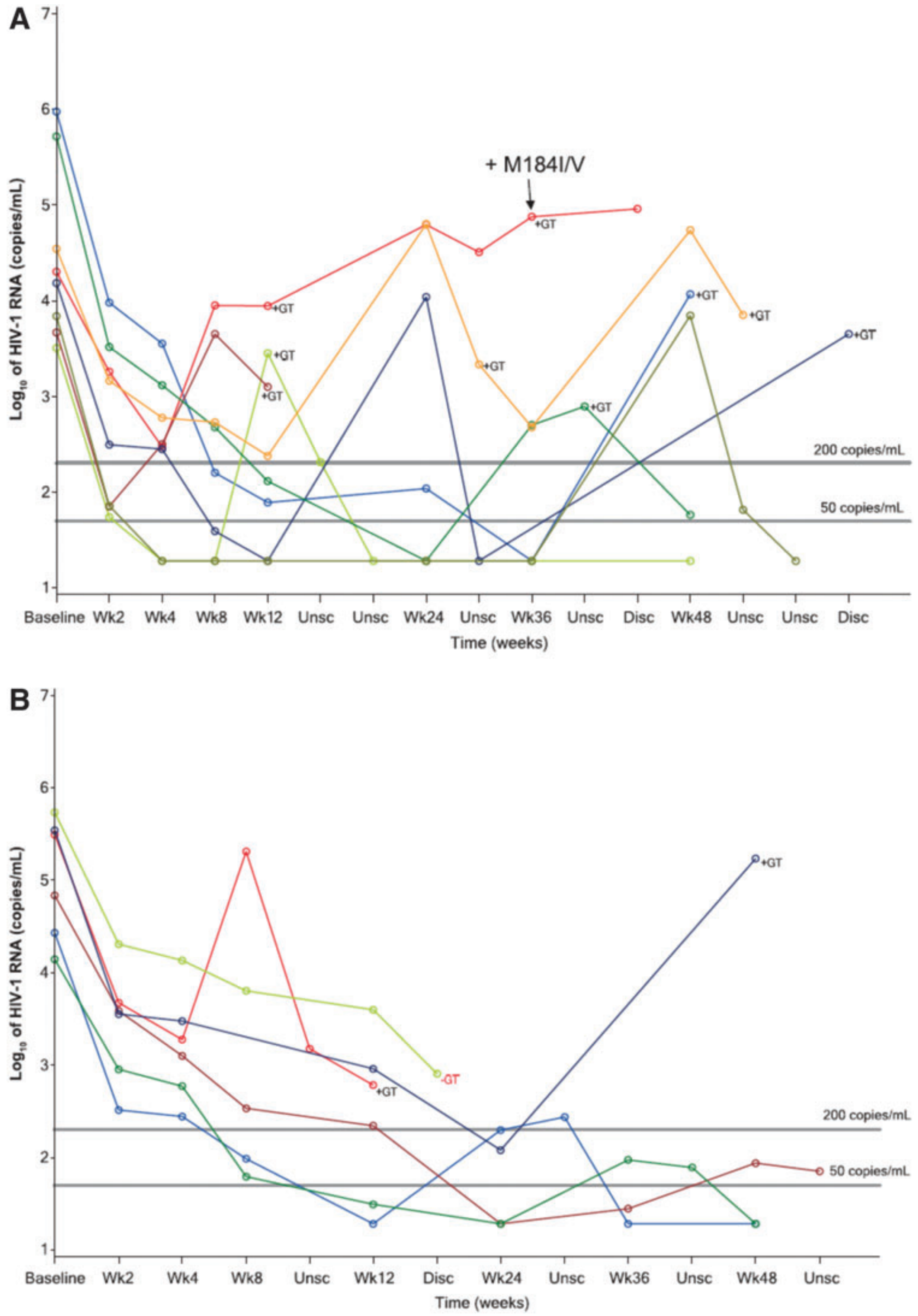

SUPPLEMENTARY FIG. S1. AMBER: Individual viral load profiles for participants with PDVF; Intent-to-treat population. **(A)** D/C/F/TAF 800/150/200/10 mg once daily (8/362 participants, 2% with PDVF). **(B)** Control regimen (D/C + F/TDF; 6/363 participants, 2% with PDVF).

PDVF, protocol-defined virologic failure; D/C/F/TAF, darunavir/cobicistat/emtricitabine/tenofovir alafenamide; Control regimen (D/C + F/TDF), darunavir/cobicistat plus emtricitabine/tenofovir disoproxil fumarate once daily; Unsc, unscheduled; Disc, discontinuation. +GT marks time point of genotype/phenotype; a *red* -GT indicates no genotype could be generated. One participant receiving D/C/F/TAF had the N(t)RTI RAM M184I/V at week 36.
