# Supplementary material for: Week 48 Resistance Analyses of the Once-Daily, Single-Tablet Regimen Darunavir/Cobicistat/Emtricitabine/Tenofovir Alafenamide (D/C/F/TAF) in Adults Living with HIV-1 from the Phase III Randomized AMBER and EMERALD Trials
Source: AIDS Res Hum Retroviruses. 2019 Dec 31;36(1):48–57. doi: 10.1089/aid.2019.0111 (PMC6944133; doi:10.1089/aid.2019.0111)
Supplement: Supplemental data [file Supp_Fig2.pdf]

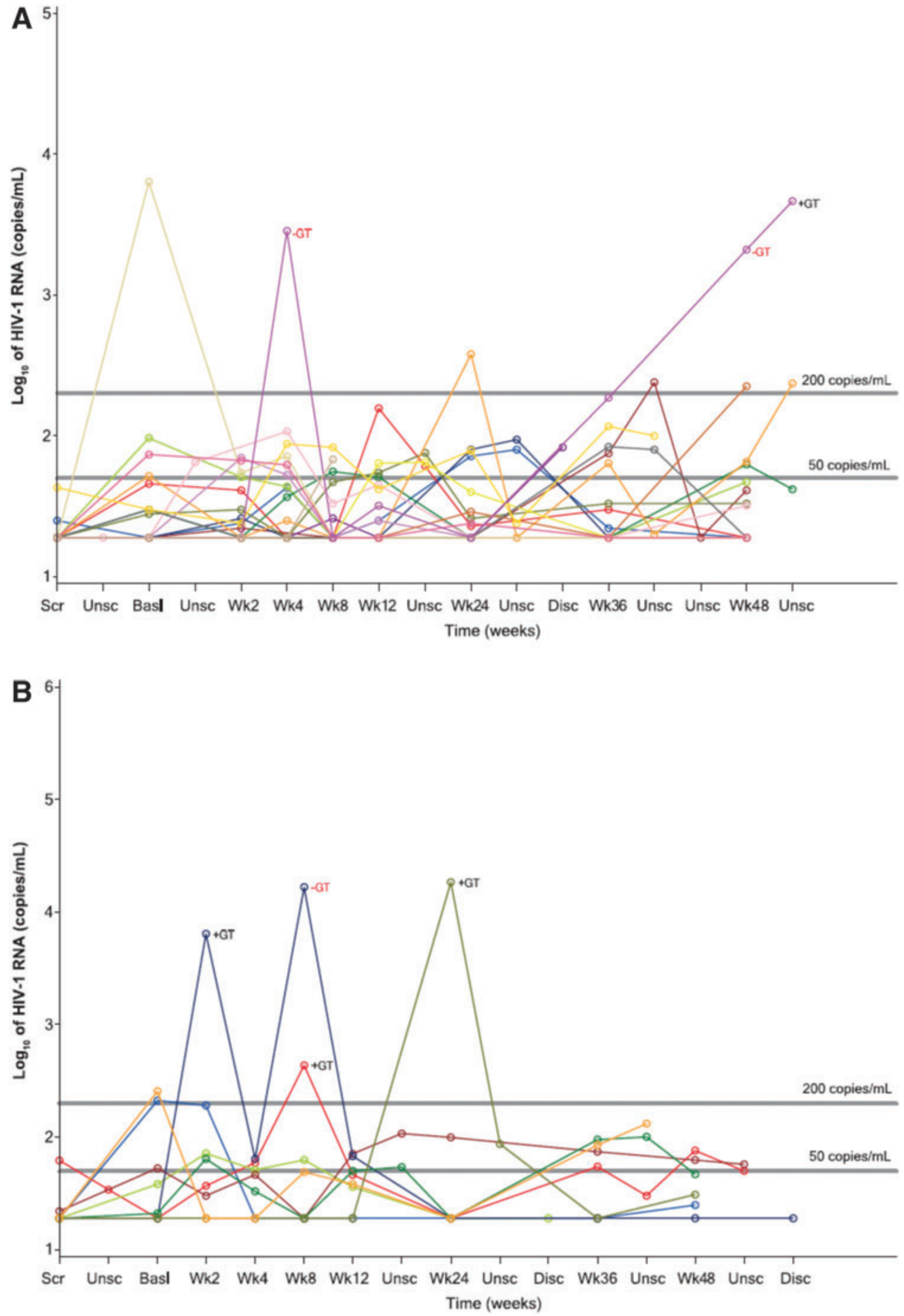

SUPPLEMENTARY FIG. S2. EMERALD: Individual viral load profiles for participants with PDVR (≥50 copies/mL); Intent-to-treat population. **(A)** D/C/F/TAF 800/150/200/10 mg once daily (19/763 participants, 2% with PDVR). **(B)** Control regimen (bPI + F/TDF; 8/378 participants, 2% with PDVR).
PDVR, protocol-defined virologic rebound; D/C/F/TAF, darunavir/cobicistat/emtricitabine/tenofovir alafenamide; Control regimen (bPI + F/TDF), boosted protease inhibitor plus emtricitabine and tenofovir disoproxil fumarate; Scr, screening; Unsc, unscheduled; Basl, baseline; Disc, discontinuation. +GT marks time point of genotype/phenotype; a *red*-GT indicates no genotype could be generated.
